# Supplementary material for: 2-Deoxy-D-glucose couples mitochondrial DNA replication with mitochondrial fitness and promotes the selection of wild-type over mutant mitochondrial DNA
Source: Nat Commun. 2021 Dec 6;12:6997. doi: 10.1038/s41467-021-26829-0 (PMC8648849; doi:10.1038/s41467-021-26829-0)
Supplement: Supplementary file 1 — Supplementary information. [file 41467_2021_26829_MOESM1_ESM.pdf]

2-Deoxy-D-glucose couples mitochondrial DNA replication with mitochondrial fitness and promotes the selection of wild-type over mutant mitochondrial DNA

Supplementary Information, Table, and Figures

1. Table of Contents
Case reports

2. Supplementary Table1
Primers used in the study.

3. Supplementary figures
3.1 Supplementary Figure 1. Effects of glucose analogues on mutant load, glycolysis, OXPHOS proteins, and mtDNA copy number.
3.2 Supplementary Figure 2. Effects of 2DG on cell growth and survival.
3.3 Supplementary Figure 3. Glucose analogues inhibit mtDNA replication and autophagy in fibroblasts carrying high levels of m.3243A>G; and replication is restored after long-term treatment.
3.4 Supplementary Figure 4. The glycolytic inhibitor KA does not inhibit mtDNA synthesis in m.3243A>G fibroblasts.
3.5 Supplementary Figure 5. Galactose has a modest inhibitory effect on mtDNA replication in m.3243A>G, while it causes extensive cell death within 48 hours.
3.6 Supplementary Figure 6. Combined glutamine and glucose restriction mimics the inhibitory effect of 2DG on cell growth in m.3243A>G mutant and control cells.
3.7 Supplementary Figure 7. 2DG induced ER-stress is higher in control cells than those carrying m.3243A>G and is alleviated by mannose supplementation, which does not prevent positive selection of wild-type mtDNA.
3.8 Supplementary Figure 8. Intermittent treatment regimes.
3.9 Supplementary Figure 9. FACS analysis.
3.10 Supplementary Figure 10. Individual experimental data.

Case Reports

Patient 1 was a 33 year-old male who first presented at the age of 14 years with a fluctuating encephalopathy and electroencephalographic evidence of focal status epilepticus involving the left posterior quadrant. Although the initial episode resolved spontaneously and completely, over subsequent years he experienced multiple stroke-like episodes with associated encephalopathy, focal motor seizure activity and disturbance of his vision and hearing with a concomitant decline in his cognitive and motor function. A genetic diagnosis of m.3243A>G (54% heteroplasmy) was established from a blood sample taken shortly after his first presentation. Cranial magnetic resonance imaging on numerous occasions demonstrated stroke-like episodes with typical T2 signal abnormality, in parieto-occipital and subsequently temporo-parietal areas, not restricted to vascular territories. Although functioning at a low level over the last few years of his life he retained awareness of his environment and had limited verbal communication. Progressive cardiomyopathy contributed to his death from lower respiratory tract infection.

Patient 2 is female, harbours the m.3243A>G mutation and was 66 years of age at the time of the skin biopsy. She was born at term following a normal pregnancy and reached all developmental milestones with delays. During adolescence she suffered with myalgia and muscle fatigue and was poor at sports at school, and is now unable to walk 200 m. She developed sensorineural hearing loss in early 30s, and was diagnosed with diabetes in early 40s, without requirement of insulin. She has subsequently been diagnosed with pigmentary retinopathy, irritable bowel syndrome and, most recently, cognitive disturbance. There is a past medical history of migraine but no suggestion of seizures, stroke-like episodes or cardiomyopathy. Neurological examination shows pigmentary retinopathy, mild ophthalmoparesis in upgaze, without ptosis, and proximal muscle weakness, predominantly affecting the lower limbs.

Table S1. Primers used in the study

Gene	mtDNA map position	Sequences 5´-3´
16SRNA	3199-3221	TTATACCCACACCCACCCAAGAA
MTND1	3330-3353	GCGATTAGAATGGGTACAATGAGG
tRNA^{LeuUUR}	3244-3258	ATGCGATTACCGGGC*
16SRNA	3202-3221	TACCCACACCCACCCAAGAA
MTND1	3308-3328	GTAGGAGGTTGGCCATGGGT
12SRNA	1156-1177	CTTAAAACTCAAAGGACCTGGC
16SRNA	1703-1725	GTTTGGCTAAGGTTGTCTGGTAG
16SRNA	2966-2989	TCAACAATAGGGTTTACGACCTCG
MTND1	3549-3572	AGGGGGGTTCATAGTAGAAGAGCG
COX2	7774-7793	CGTCTGAACTATCCTGCCCG
COX2	7855-7874	TGGTAAGGGAGGGATCGTTG
APP1	Not applicable	TTTTTGTGTGCTCTCCCAGGTCT
APP1	Not applicable	TGGTCACTGGTTGGTTGGC

* primer used for the final pyrosequencing step

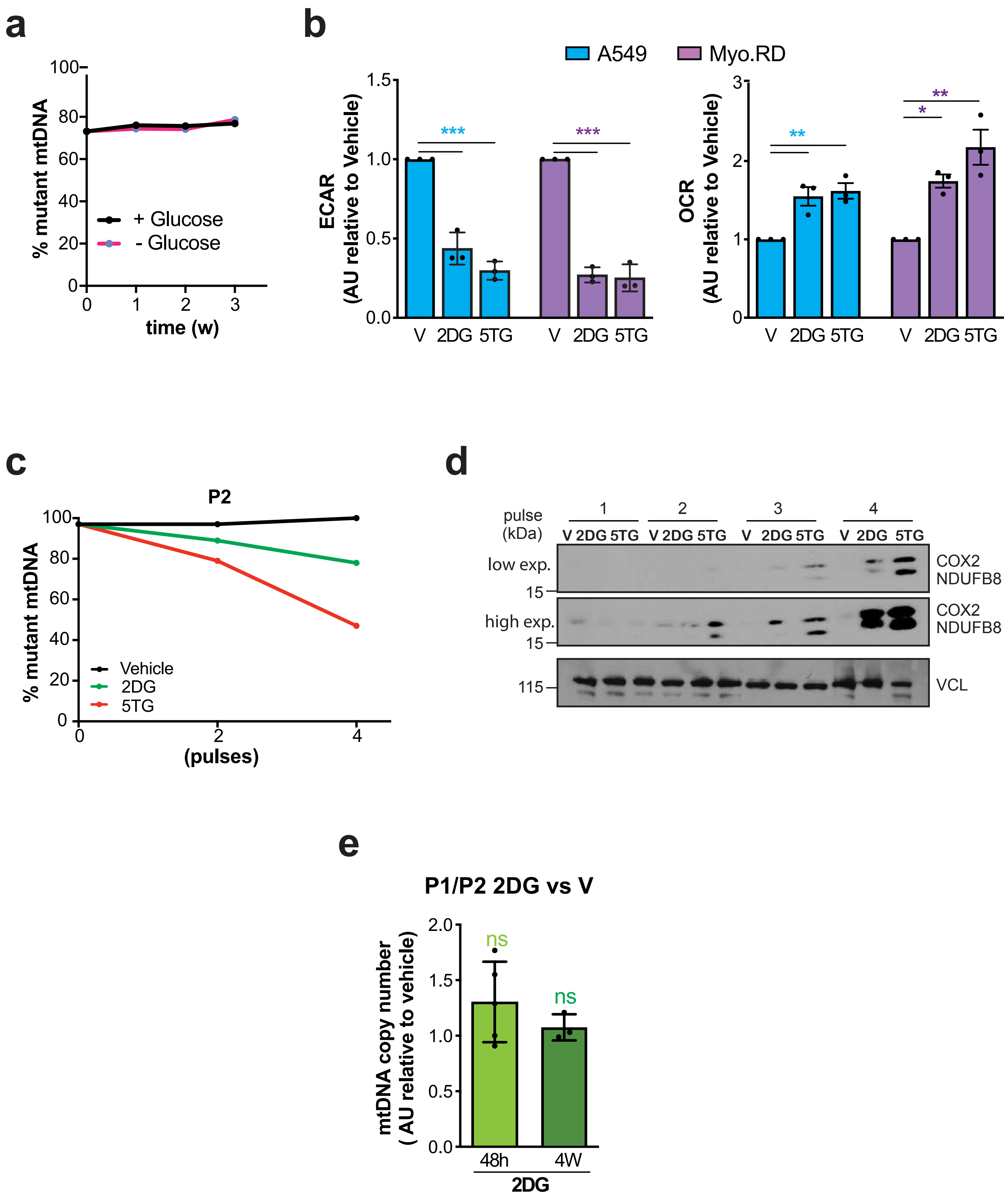

Supplementary Figure 1

Supplementary Figure 1. Effects of glucose analogues on mutant load, glycolysis, OXPHOS proteins, and mtDNA copy number. **a**, A549 cells carrying 76% m.3243A>G were grown, in DMEM supplemented with 0 (red line) or 25 mM (black line) glucose and 10% FBS. DNA was harvested at intervals and the mutant load determined by pyrosequencing (see methods) and plotted against time. **b**, The effects of 2DG and 5TG on the extracellular acidification rate (ECAR) and on the Oxygen consumption rate (OCR) were measured using a XF flux-analyzer (Seahorse instrumentation) in A549 and Myo.RD by injecting the compounds directly on the plate through one of the ports of the cartridge. Based on the assay, glycolysis was inhibited by 60-80% by the compounds. Data are derived from 3 independent experiments for each cell line and errors are ± SD. One-way ANOVA: ****P*(A549 Veh vs 2DG ECAR)=0.000123; ****P*(A549 Veh vs 5TG ECAR)=0.000034; ****P*(Myo.RD Veh vs 2DG ECAR)=0.00001; ****P*(Myo-RD Veh vs 5TG ECAR)=0.000008; ***P* (A549 Veh vs 2DG OCR)=0.0087; ***P*(A549 Veh vs 5TG OCR)=0.0049; **P*(Myo.RD Veh vs 2DG OCR)=0.0156; ***P*(Myo.RD Veh vs 5TG OCR)=0.0017. **c**, P2 fibroblasts were treated intermittently with or without 2DG or 5TG and the level of heteroplasmy was measured by pyrosequencing and the proportion of mutant mtDNA plotted against time. **d**, The progressive increase in wild-type mtDNA associated with 2DG or 5TG treatment (c) is accompanied by increasing amounts of OXPHOS proteins, based on Western blots analysis of the OXPHOS subunits COX2 (complex IV) and NDUFB8 (complex I) in whole cell lysates at the indicated time-points. Vinculin (VCL) is shown as loading control; the same progressive increase in OXPHOS proteins was observed in two additional independent experiments. **e**, Heteroplasmy changes were not associated with any significant alteration in mtDNA copy number in fibroblasts treated with 2DG. Data represent the mean value ±SD of n=2 and 3 independent experiments for P1 and P2, respectively. One-way ANOVA: ns *P*(P1/P2 Veh vs 2DG 48h)=0.1213; ns *P*(P1/P2 Veh vs 2DG 4w)=0.8729. Source data are provided as a Source Data file.

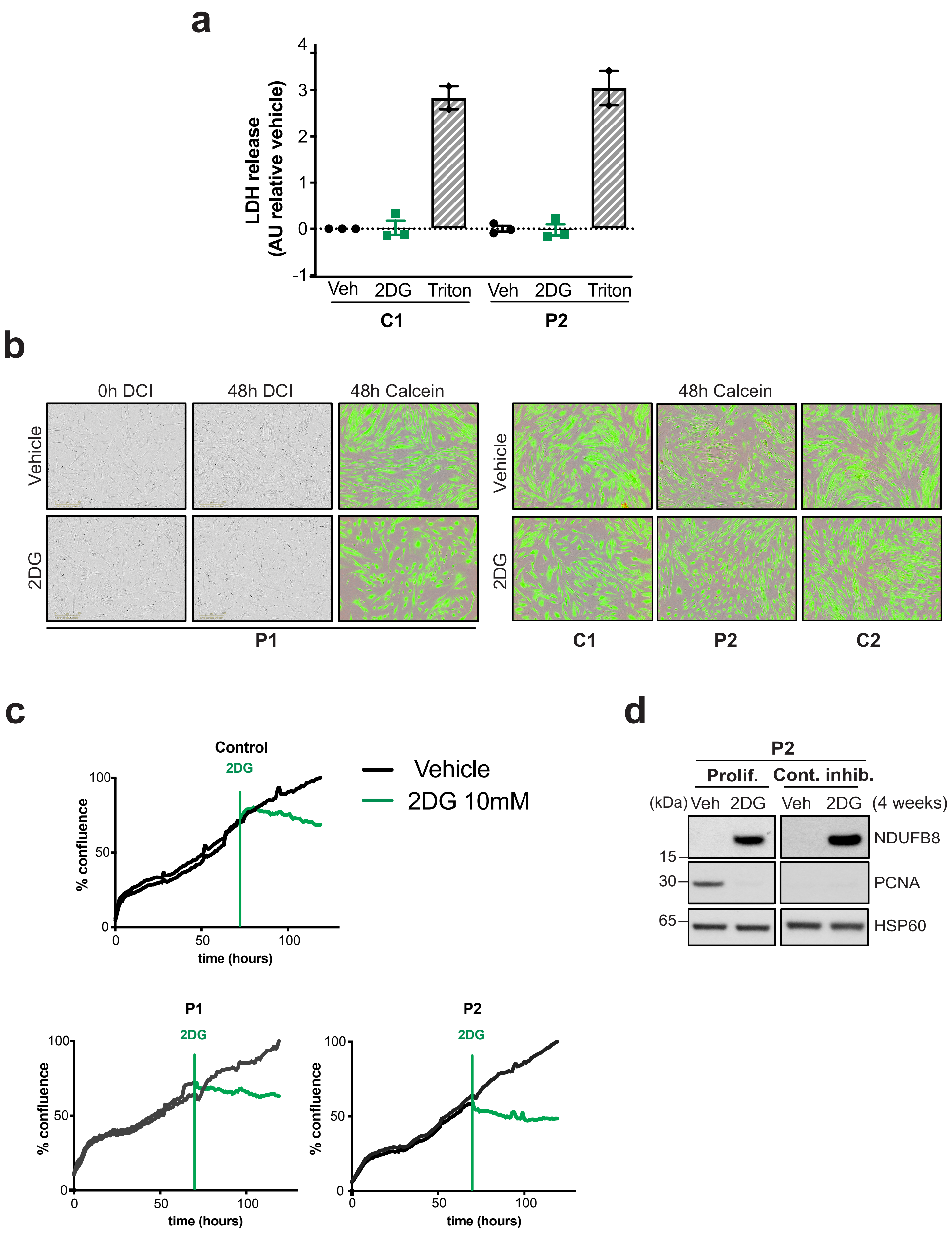

Supplementary Figure 2

Supplementary Figure 2. Effects of 2DG on cell growth and survival. a, **b**, 2DG did not cause an increase in cell death, based on visual detection of cellular debris or detached cells, or calcein labelling of viable fibroblasts after 48 h exposure to 2DG, or LDH levels in spent medium harvested from control and patient-derived fibroblasts after treating with or without 2DG for 48 h, using as control cells lysed with Triton X-100; n=3 independent experiments. Bar = 300 μm. **c**, 2DG inhibits the growth of fibroblasts with mutant and wild-type mtDNA, based on proliferation rate determined using an Incucyte™-adapted incubator. Cells were imaged every hour and the proliferation rate was determined by analysing the sequence of images with the manufacturer's software to generate growth curves expressing cell density over time. The start of the 2DG treatment is indicated by the green bar; vehicle - black line and 2DG-treated cells - green line. **d,** Mutant cells (P2) treated with 2DG or vehicle in proliferating or contact inhibition conditions for 4 weeks. Levels of NDUFB8 are increased in 2DG treated samples without an increase of mitochondrial mass (HSP60). PCNA, a marker of cell proliferation, is markedly down-regulated in the 2DG-treated samples; n=3 and 2 independent experiments in contact inhibition conditions for 2DG and 5TG, respectively. Source data are provided as a Source Data file.

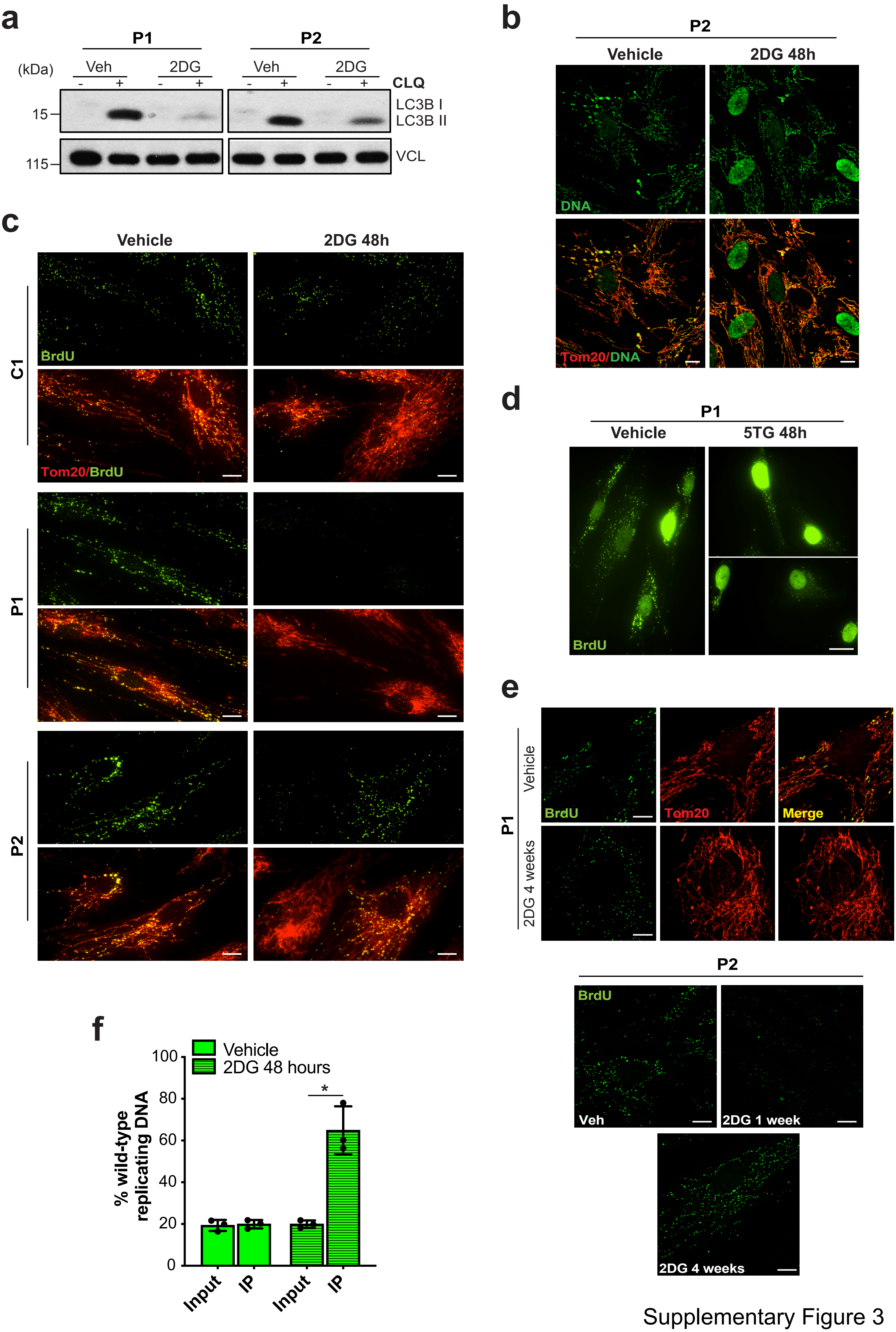

Supplementary Figure 3

Supplementary Figure 3. Glucose analogues inhibit mtDNA replication and autophagy in fibroblasts carrying high levels of m.3243A>G; and replication is restored after long-term treatment.

a, 2DG inhibits autophagy in cells with high mutant load; the inhibitory effect is greater in P1 (92% m.3243A>G) than P2 (85% m.3243A>G) fibroblasts. Cells were treated with or without 10 mM 2DG for 48 h, and with or without CLQ for the final 6 hours. Extracted proteins were immunolabelled for the lysosomal marker LC3B, with vinculin (VCL) as the loading control; n = 4 independent experiments. **b**, P2 fibroblasts treated with vehicle or 10 mM 2DG or 48 h immunostained for DNA (green) and the mitochondrial network (TOM20, red); n = 3 independent experiments. Bar = 10 µm. **c**, Representative images (widefield microscopy) of P1, P2 and C1 fibroblasts treated with vehicle or 2DG and immunostained for BrdU (green) and TOM20 (red); n=6 independent experiments. Bar = 20 µm. **d**, P1 fibroblasts treated with vehicle or 10 mM 5TG or 48 h and immunostained for BrdU (green); n = 6 independent experiments. Bar = 20 µm. **e**, Representative images (confocal microscopy) showing BrdU labelling in P1 cells treated with vehicle (that does not inhibit mtDNA synthesis) or 2DG for 4 weeks, with the BrdU present for the final 13 h of a 48 h period of exposure to 10 mM 2DG. Bar = 10 µm. P2 cells treated with vehicle for 1 week; 2DG for 1 week or 2DG for 4 weeks, as indicated. Bar = 15 µm. **f**, Individual data points of the BrdU-DNA immunoprecipitations (IP) described in Fig. 3e. Plots represent the proportion of wild-type mtDNA before (Input) and after (IP) capture by the anti-BrdU antibody from P2 cells, treated with or without 2DG. Data represent the mean value ±SEM of n = 3 independent experiments. Unpaired, two-sided t-test with Welsh's correction *P(Veh vs 2DG IP 48h)=0.019. Source data are provided as a Source Data file.

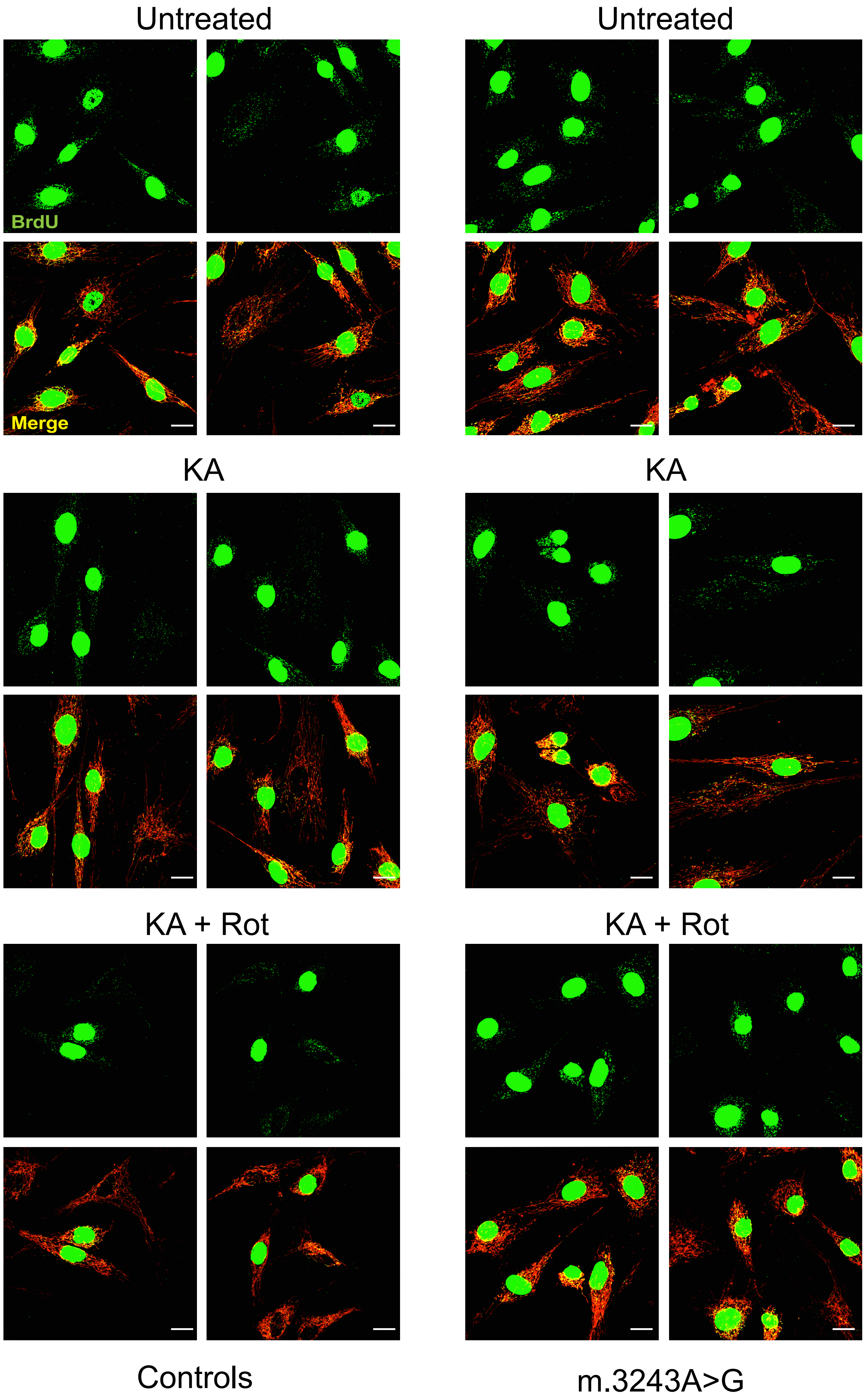

Supplementary Figure 4

Supplementary Figure 4. The glycolytic inhibitor KA does not inhibit mtDNA synthesis in m.3243A>G fibroblasts. Representative images of Control (C1 and C2) and Patient (P1 and P2) fibroblasts grown for 24 h in medium with and without 0.5 μM koningic acid (KA) and where indicated 1 μM rotenone (rot). For the final 13 h 50 μM BrdU was included in the medium after which the cells were fixed and stained with anti-BrdU antibody – green signal and merged with anti-Tom20 (red – mitochondrial network); n = 4 independent experiments. Bar = 20 μm.

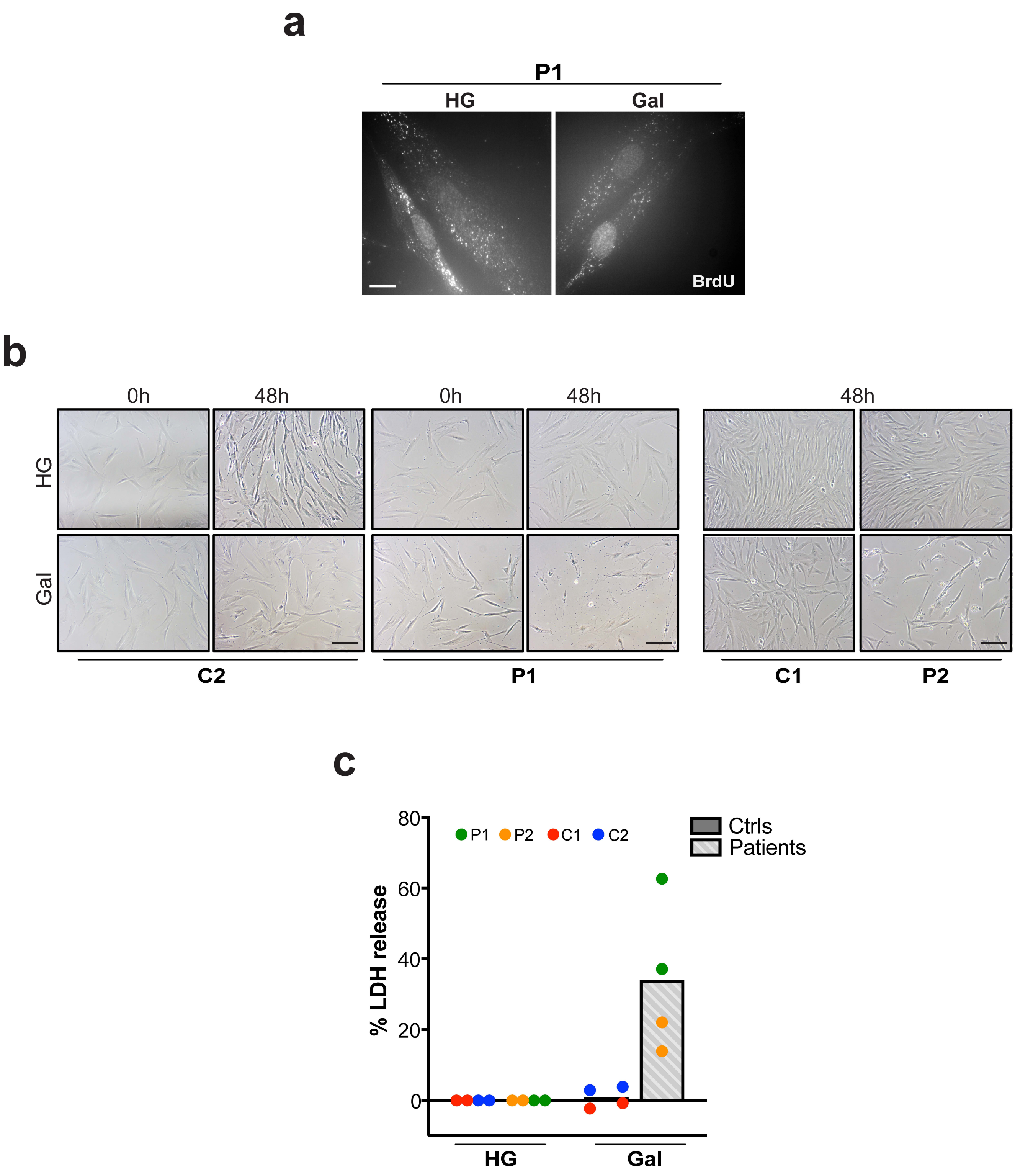

Supplementary Figure 5

Supplementary Figure 5. Galactose has a modest inhibitory effect on mtDNA replication in m.3243A>G, while it causes extensive cell death within 48 hours. **a**, In contrast to 2DG and 5TG, substitution of 25 mM glucose (HG) with 5 mM galactose (Gal) does not inhibit mtDNA synthesis based on BrdU labelling in P1 fibroblasts, n = 4 independent experiments. Bar = 20 µm. Replacing glucose with galactose lead to **b**, many fewer cells after 48h (n = 4 independent experiments), a good part of which was owing to **c**, cell death evidenced by elevated LDH (values expressed as a proportion of 100% cell lysis with Triton-X 100). n = 2 independent experiments for each cell line (C1, C2, P1 and P2). Bar = 100 µm. Source data are provided as a Source Data file.

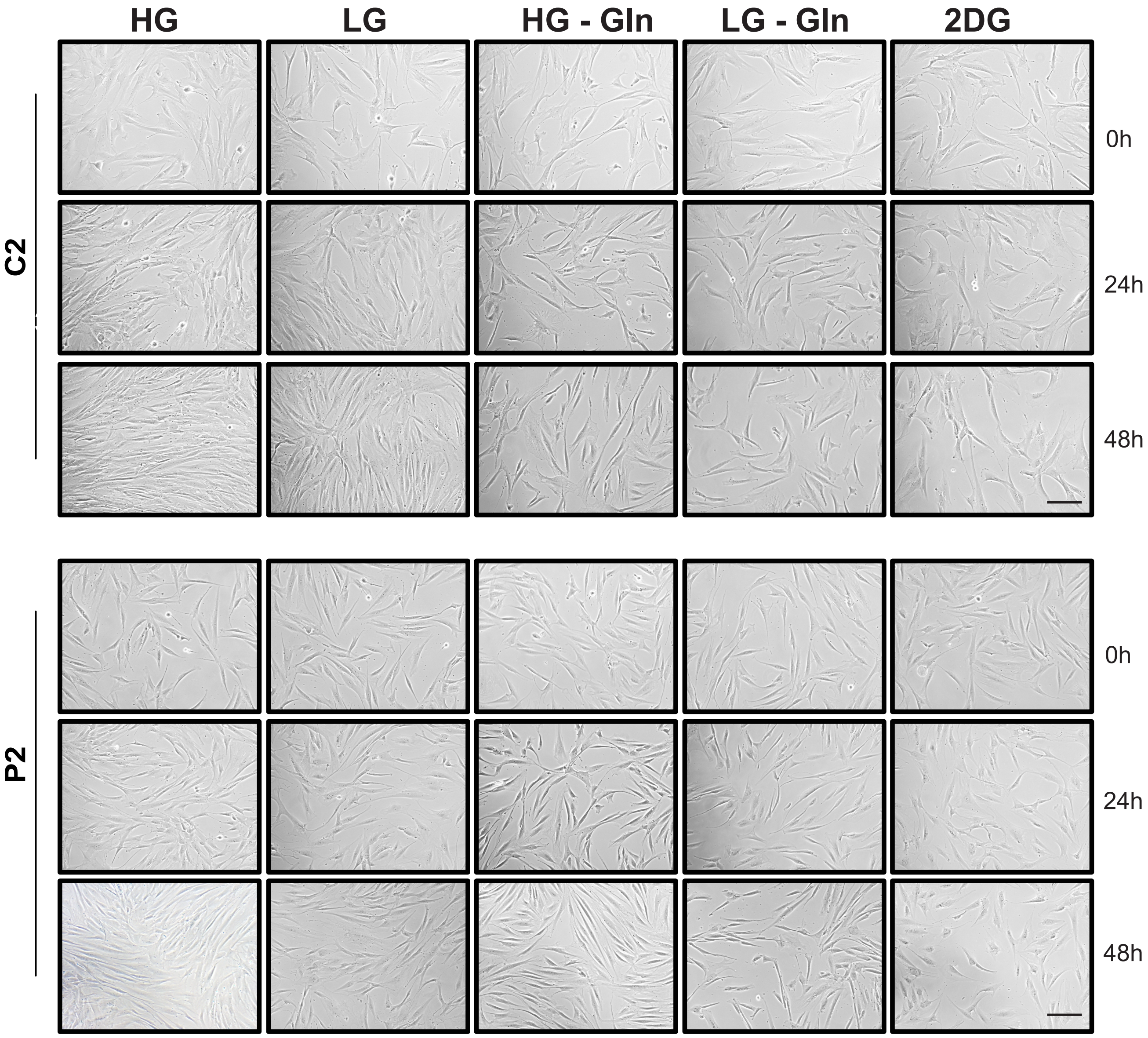

Supplementary Figure 6

Supplementary Figure 6. Combined glutamine and glucose restriction mimics the inhibitory effect of 2DG on cell growth in m.3243A>G mutant and control cells. Representative images of Control (C2) and Patient (P2) fibroblasts grown for 48 h in medium containing, or lacking, glutamine, and different concentrations of glucose for 48 h, or 10 mM 2DG. While glucose restriction slows the growth of mutants but not control cells, additional withdrawal of glutamine markedly inhibits the cell growth in both mutant and control, as does 2DG. HG, 25 mM glucose; LG, 1 mM glucose; - Gln, no glutamine. n = 3 independent experiments. Bar = 100 μm.

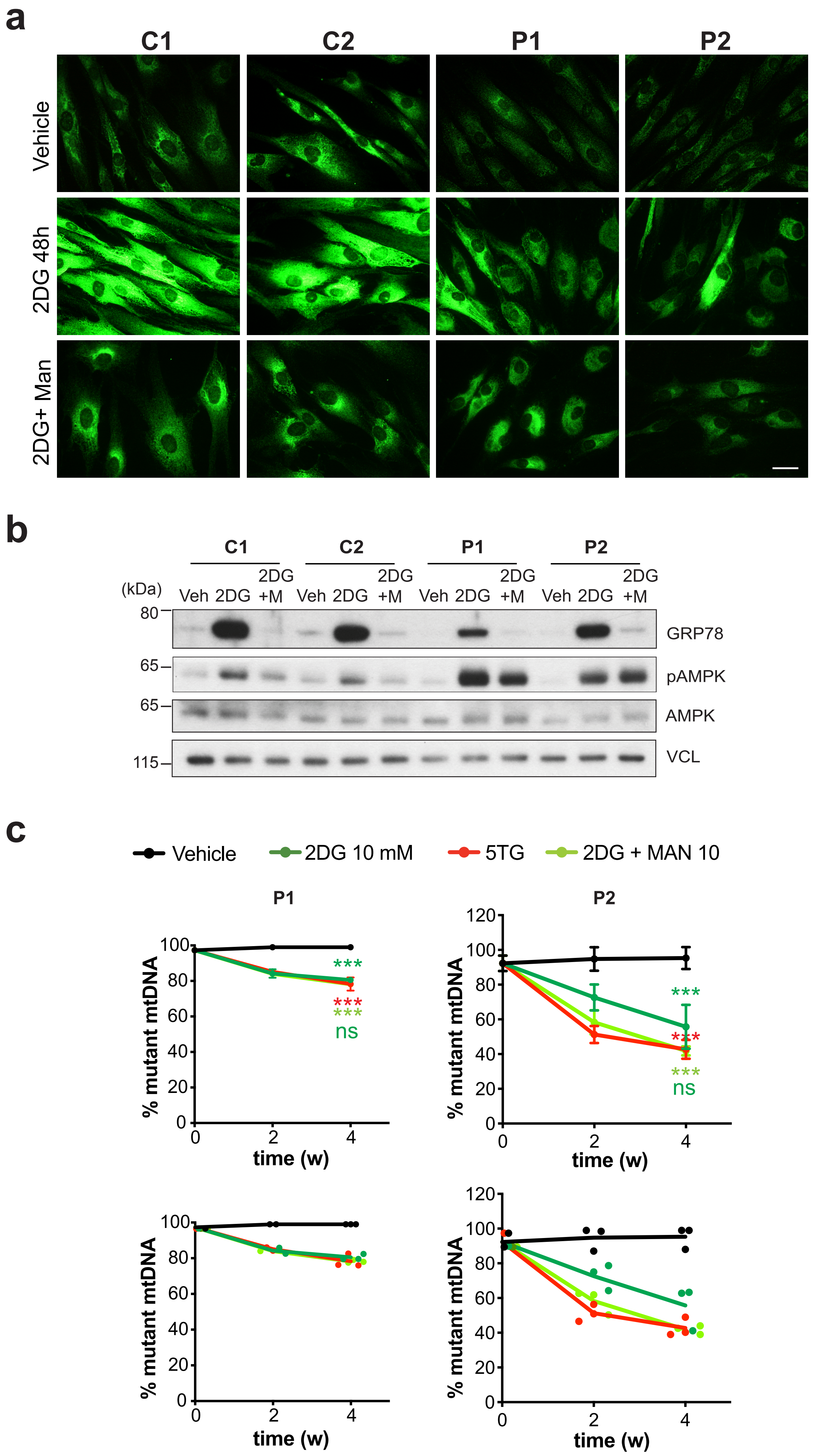

Supplementary Figure 7

Supplementary Figure 7. 2DG induced ER-stress is higher in control cells than those carrying m.3243A>G and is alleviated by mannose supplementation, which does not prevent positive selection of wild-type mtDNA. a, GRP78 was detected by immunofluorescence in control fibroblasts (C1, C2) and fibroblasts carrying m.3243A>G (P1 and P2), treated with and without 10 mM 2DG for 48 h in the presence and absence of mannose (M); n = 5 independent experiments. Bar = 50 μm. **b**, Control (C1, C2) and mutant fibroblasts (P1, P2) treated with 10 mM 2DG with or without 10 mM mannose (M) for 48 h, or treated with vehicle. 5 μM choloroquine (CLQ) was added for the final 6 hours of the incubation where indicated to block autophagy. Cellular proteins, GRP78, AMPK, pAMPK and the loading control vinculin (VCL) were detected by immunoblotting; n = 3 independent experiments. **c**, P2 and, P1 cells were subjected to 8 pulses of vehicle, 5TG or 2DG with and without mannose, over the course of 4 weeks and the mutant load assayed every 2 weeks. Data represent the mean value ±SD of n = 3 independent experiments. One way ANOVA: ****P*(P1 Veh vs 2DG 4w)= 0.000021; ****P*(P1 Veh vs 5TG 4w)= 0.000009; ****P*(P1 Veh vs 2DG+ Man 4w)= 0.000008; ns *P*(P1 2DG vs 2DG+ Man 4w)= 0.540348; ***P*(P2 Veh vs. 2DG 4w)= 0.00104; ****P*(P2 Veh vs. 5TG 4w)= 0.000144; ****P*(P2 Veh vs 2DG + Man 4w)= 0.000127; ns *P*(P2 2DG vs 2DG+ Man 4w)= 0.198559. Duplicate charts showing individual data points are showed immediately below. Data points to the 2DG & 5TG treatments are also included in main Fig. 1e and 1f. Source data are provided as a Source Data file.

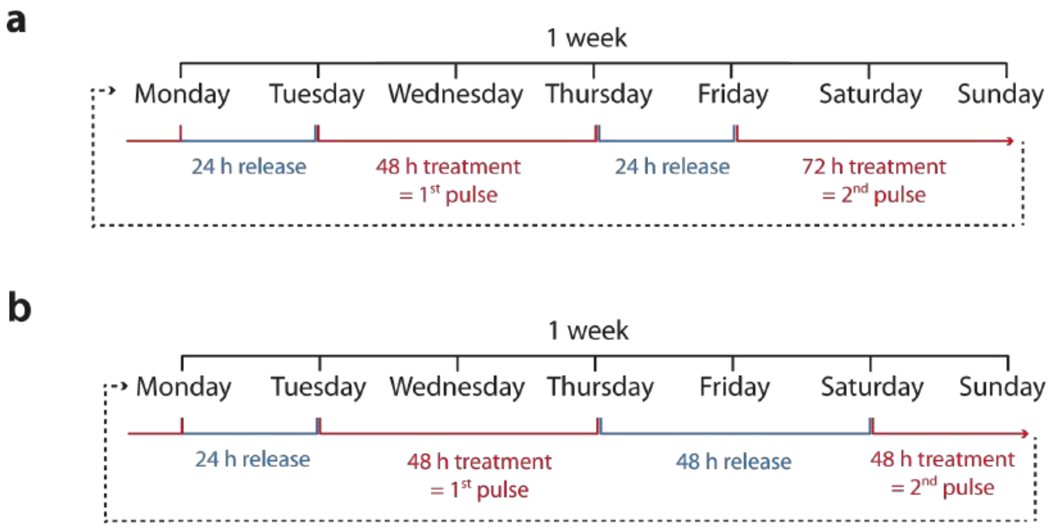

Supplementary Figure 8. Intermittent treatment regimes. a, The treatment regime for A549 and RD cybrids comprised weekly cycles of 48 h in the presence of drug or modified medium (first pulse) followed by 24 h without drug or non-restrictive medium (release), 72 h with drug or modified medium (second pulse) and a further 24 h recovery, i.e. two pulses per week. **b**, m.3243A>G fibroblasts treatment involved two pulses of 48 h separated by 24 or 48 h without drug or modified treatment (release).

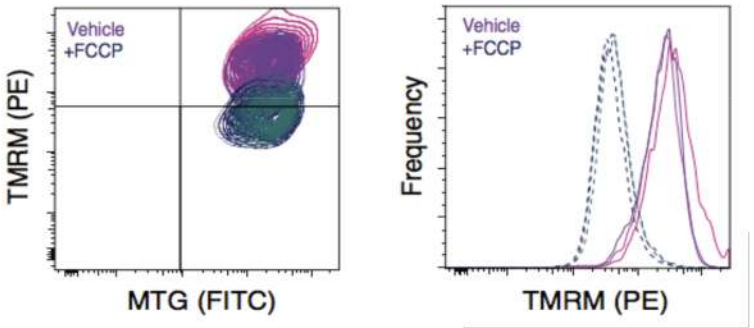

Supplementary Figure 9. FACS analysis. TMRM labels only live cells: a representative plot indicates the gating strategy. FCCP was used to generate a profile corresponding to 100% depolarized cells.

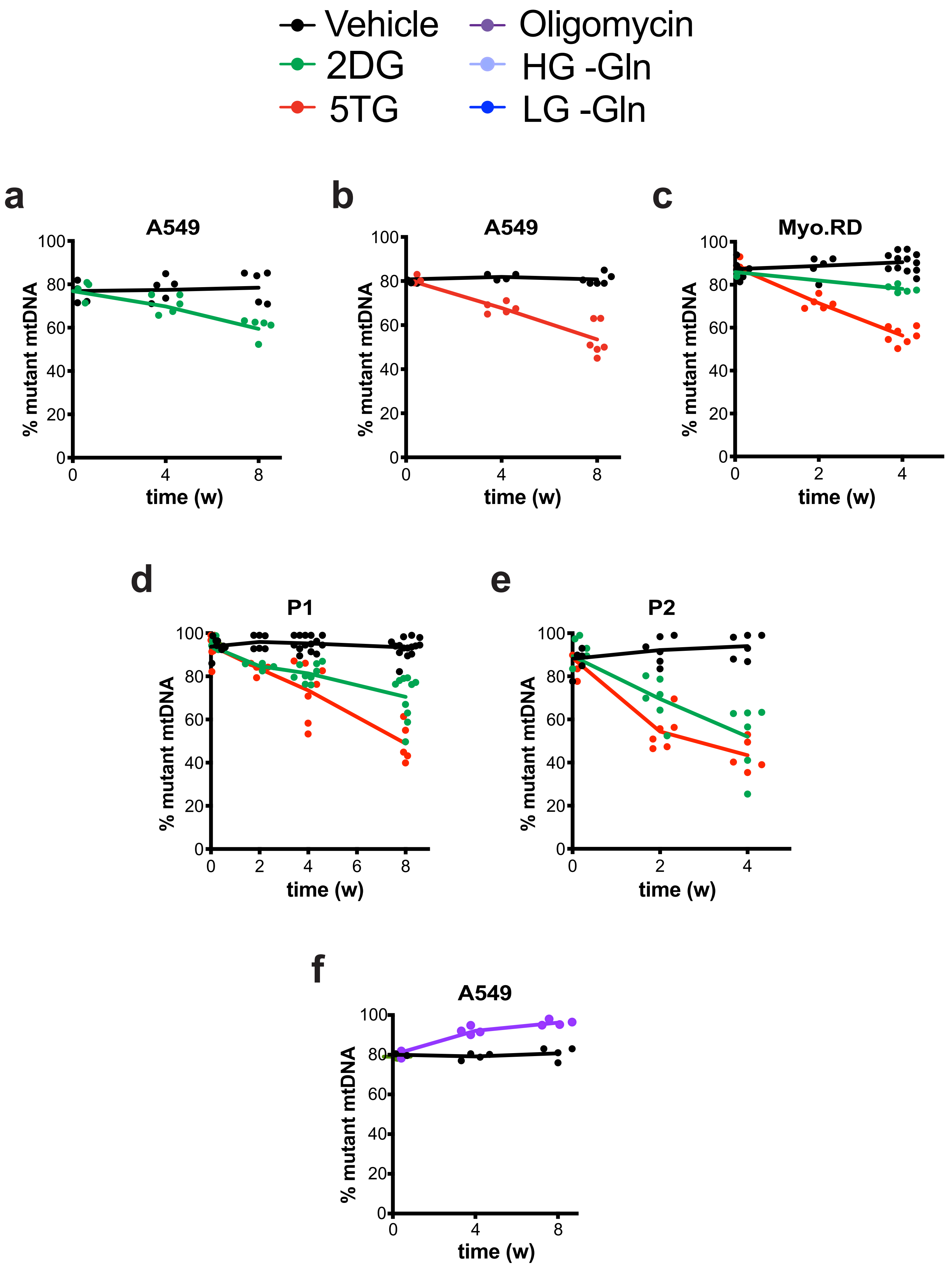

Supplementary Figure 10

Supplementary Figure 10. Individual experimental data. Individual replicates with means connected are showed for some panels of the Figures 1 and 4. Panel **a** corresponds to Fig. 1a; **b** to Fig. 1c; **c** to Fig.1d; **d** to Fig. 1e; **e** to Fig.1f; **f** to Fig. 4h. Source data are provided as a Source Data file.
