# Supplementary material for: 2-Deoxy-D-glucose couples mitochondrial DNA replication with mitochondrial fitness and promotes the selection of wild-type over mutant mitochondrial DNA
Source: Nat Commun. 2021 Dec 6;12:6997. doi: 10.1038/s41467-021-26829-0 (PMC8648849; doi:10.1038/s41467-021-26829-0)
Supplement: Supplementary file 3 — Reporting summary. [file 41467_2021_26829_MOESM3_ESM.pdf]

nature research

Corresponding author(s): Antonella Spinazzola

Last updated by author(s): Oct 16, 2021

Reporting Summary

Nature Research wishes to improve the reproducibility of the work that we publish. This form provides structure for consistency and transparency in reporting. For further information on Nature Research policies, see our Editorial Policies and the Editorial Policy Checklist.

Statistics

For all statistical analyses, confirm that the following items are present in the figure legend, table legend, main text, or Methods section.

n/a	Confirmed	
☐	☒	The exact sample size (*n*) for each experimental group/condition, given as a discrete number and unit of measurement
☐	☒	A statement on whether measurements were taken from distinct samples or whether the same sample was measured repeatedly
☐	☒	The statistical test(s) used AND whether they are one- or two-sided<br>*Only common tests should be described solely by name; describe more complex techniques in the Methods section.*
☒	☐	A description of all covariates tested
☒	☐	A description of any assumptions or corrections, such as tests of normality and adjustment for multiple comparisons
☐	☒	A full description of the statistical parameters including central tendency (e.g. means) or other basic estimates (e.g. regression coefficient) AND variation (e.g. standard deviation) or associated estimates of uncertainty (e.g. confidence intervals)
☐	☒	For null hypothesis testing, the test statistic (e.g. F, t, r) with confidence intervals, effect sizes, degrees of freedom and P value noted<br>*Give P values as exact values whenever suitable.*
☒	☐	For Bayesian analysis, information on the choice of priors and Markov chain Monte Carlo settings
☒	☐	For hierarchical and complex designs, identification of the appropriate level for tests and full reporting of outcomes
☒	☐	Estimates of effect sizes (e.g. Cohen's *d*, Pearson's *r*), indicating how they were calculated

Our web collection on statistics for biologists contains articles on many of the points above.

Software and code

Policy information about availability of computer code

Data collection	Confocal images were acquire via a a SP5 TCS Inverted Confocal Microscope (Leica Biosystem) or Nikon Ti Inverted Confocal microscope (Nikon). Laser power, gain and offset parameters were kept constant for each experiment. Cell number was measured via an Incucyte Zoom Instrument (Essen Bioscience).<br>Pyrosequencing data were obtained using a PSQ 96MA pyrosequencer (Biotage/Qiagen).<br>For TMRM, see Flow Cytometry.<br>Western blots were digitalized using a Canoscan 9000F scanner (Canon).
Data analysis	The microscope software for Leica was Leica Application Suite X, with the file extension ".lif" format, whereas for the Nikon microscope we used NIS Element Software in a ".nd2" format. Confocal image analysis used the plugins available with Fiji imageJ (2.0.0-rc-15/1.49h), with equal linear adjustments to all images in any comparison.<br>Microsoft Excel (14.4.8) and GraphPad Prism (versions 7 and 8) were used for data storage and production of charts. Cell growth was calculated using Incucyte Zoom software 2015A. Pyrosequencing data analysis used SQ 96 MA 2.1 software for generation of pyrograms, pyrogram quality control and automatic allelic quantification. Optical density quantification of bands detected by Western blotting was carried out using the designated tools available with Fiji ImageJ (2.0.0-rc-15/1.49h).

For manuscripts utilizing custom algorithms or software that are central to the research but not yet described in published literature, software must be made available to editors and reviewers. We strongly encourage code deposition in a community repository (e.g. GitHub). See the Nature Research guidelines for submitting code & software for further information.

Data

Policy information about availability of data

All manuscripts must include a data availability statement. This statement should provide the following information, where applicable:

- Accession codes, unique identifiers, or web links for publicly available datasets
- A list of figures that have associated raw data
- A description of any restrictions on data availability

All the data supporting the study are available within the paper and its supplementary information files. Source data are provided with this paper.

Field-specific reporting

Please select the one below that is the best fit for your research. If you are not sure, read the appropriate sections before making your selection.

☒ Life sciences ☐ Behavioural & social sciences ☐ Ecological, evolutionary & environmental sciences

For a reference copy of the document with all sections, see nature.com/documents/nr-reporting-summary-flat.pdf

Life sciences study design

All studies must disclose on these points even when the disclosure is negative.

Sample size	Very few primary cell lines are available from patients with a specific mitochondrial disease, even the relatively common one of m.3243A>G. We obtained 2 fibroblast lines from two different centres and performed many separate experiments (>30 in total); additionally, we studied the same mutant mitochondrial DNA (from a third individual with the disease) in two cancer cell lines.
Data exclusions	Appreciable cell death (by visual inspection) was an exclusion criteria for the segregation experiments - as it would result in inter-cellular selection, whereas we were interested only in segregation that could be ascribed to intra-cellular selection. Thus, one compound did reproducibly result in an increase in wild-type mitochondrial DNA, but also caused cell death and so is not reported in the study. Whenever tested, the compounds produced no detectable increase in cell death in the mutant cells (e.g. Supplementary Fig. 2a).
Replication	Individual data points are shown in the main or supplementary figures showing reproducibility; each data point was a separate experiment (not a technical replicate). Notably, four researchers (and one PI) carried out segregation experiments at different times and all observed that the compounds induced segregation to wild-type mitochondrial DNA (the key finding of the study). Other key findings, such as the inhibition of mtDNA replication and autophagy, the effects of the compounds on ER-stress and the effect of the glutamine and glucose restriction (and glycolysis) were demonstrated by four researchers in independent experiments.
Randomization	The studies were in vitro and so randomization was not applicable.
Blinding	In most cases, the cells and cell derivatives were handled by a single researcher dedicated to the project and so were not blinded. In later experiments, pyrosequencing (to determine the level of mutant and wild-type mitochondrial DNA) was performed by one individual with no knowledge of the identity of the samples.

Reporting for specific materials, systems and methods

We require information from authors about some types of materials, experimental systems and methods used in many studies. Here, indicate whether each material, system or method listed is relevant to your study. If you are not sure if a list item applies to your research, read the appropriate section before selecting a response.

Materials & experimental systems

n/a	Involved in the study	
☐	☒	Antibodies
☐	☒	Eukaryotic cell lines
☒	☐	Palaeontology and archaeology
☒	☐	Animals and other organisms
☐	☒	Human research participants
☒	☐	Clinical data
☒	☐	Dual use research of concern

Methods

n/a	Involved in the study	
☒	☐	ChIP-seq
☐	☒	Flow cytometry
☒	☐	MRI-based neuroimaging

Antibodies

Antibodies used	Bromodeoxyuridine Biorad Cat.# MCA2060; dilution 1:200. (discontinued)<br>BrdU, Abcam, Cat.# ab6326 1:200.<br>DNA, Progen Cat.# AC-30-10 dilution 1:250.

GAPDH, Abcam Cat.# ab8245; dilution 1:2000.
GAPDH,Sigma, Cat.# G8795, dilution 1:10000
GRP78, Santa Cruz Biotech Cat.# sc-13968; dilution 1:1000.
HSP60, Abcam, Cat.# ab46798; dilution 1:1000.
LC3B, Sigma Cat,# L7543 1:1000
MTCO-2, Abcam Cat# ab110258; dilution 1:1000.
NDUFB8, Abcam Cat.# ab110242; dilution 1:1000.
AMPK-alpha, Cell Signaling Cat.# 2532; dilution 1:1000.
phosphoAMPK-alpha, Cell Signaling Cat.# 2535; dilution 1:1000.
TOM20, Santa Cruz Biotech Cat.# sc-11415; dilution 1:4000.
TOM20, Abcam, Cat.# ab186735,dilution 1:1000.
Vinculin, Abcam Cat.# ab18058; dilution 1:1000.
PCNA, Santa Cruz Biotech Cat.# sc-56;dilution 1:2000.
Anti-Mouse IgG (H+L), HRP Conjugate, Promega Cat.# W4021 dilution 1:4000.
Anti-Rabbit IgG (H+L), HRP Conjugate, Promega, Cat.# W4011, dilution 1:4000.
Alexa Fluor®-488 goat- anti-mouse, Invitrogen, Cat.# A-10684, dilution 1:1000.
Alexa Fluor®-568 goat- anti-mouse, Invitrogen, Cat.# A-11004, dilution 1:1000.
Alexa Fluor® 568 donkey anti-rabbit, Invitrogen, Cat.# A-10042, dilution 1:1000.
Alexa Fluor®-488 goat- anti-rat, Invitrogen, Cat.# A-11006, dilution 1:1000.

Validation

Anti-BrdU – in our experiments this rat monoclonal antibody labels cytoplasmic foci within the mitochondrial network consistent with mitochondrial DNA – and only in cells supplemented with BrdU (e.g. Supplementary Figure 3). The antibody immunoprecipitates PCR amplifiable material using primers to mitochondrial DNA (i.e. IPs mtDNA) when the culture medium has been supplemented with BrdU for 16 hours (Figure 3e). Manufacturer's website shows mouse anti Bromodeoxyuridine antibody used for the identification of proliferating cells in the rat dentate gyrus by immunofluorescence. Also validated previously, PMID: 24740847; DOI: 10.1002/glia.22671.
Anti-DNA - labels the nucleus and cytoplasmic foci within the mitochondrial network consistent with nuclear and mitochondrial DNA respectively (e.g. Figure 3b). The antibody generates a low signal in cytoplasm in known cases of mtDNA depletion, as expected, and consistent with PCR analysis of copy number. Mouse monoclonal antibody; manufacturer's website shows the antibody detects DNA in the nucleus and the mitochondria of human U2OS cells, and there is no signal in cells that lack mitochondrial DNA. Also validated previously, PMID: 28195532 PMCID: PMC5367896 DOI: 10.7554/eLife.22187.
Anti-GRP78 – protein detected has correct size and immunocytochemistry revealed it is located in the endoplasmic reticulum and induced in response to 2DG as expected (Supplementary Figure 7). Manufacturer's website shows Western blots and immunocytochemistry of human and rodent cells and proteins; cited in 434 publications, e.g. PMID: 33111362 DOI: 10.1002/ptr.6925.
Anti-LC3B – produces 2 bands of the expected size, the lower one accumulated after chloroquine treatment (Figure 3a). Manufactures' website shows western blot and immunocytochemistry analysis of the antibody in HEK, HeLa and human rhabdomyosarcoma (RD-A) cells. Xueyan Xi X, et al. The interplays between autophagy and apoptosis induced by enterovirus 71. PLoS One. 2013;8(2):e56966. PMID: 23437282.
Anti-HSP60 – in our experiments the antibody detects a protein of the predicted size in the correct cellular compartment (the mitochondria). Manufactures' website shows western blot and immunocytochemistry analysis of the antibody and indicates it is a rabbit polyclonal antibody reactive against the mouse, cow, pig and arabidopsis protein as well as that of humans. It has been cited in 98 publications, e.g. PubMed: 33144179.
Anti-TOMM20 – correct sized protein in correct cellular compartment (the mitochondria), detected cellular structure contains the cytoplasmic DNA foci (mtDNA molecules). Manufactures' website shows western blot and immunocytochemistry analysis of the antibody and indicates it is a rabbit monoclonal antibody reactive against the mouse, rat and human protein. It has been cited in 69 publications, e.g. PubMed: 32515035.
Anti-MTCO-2 – correct sized protein in correct cellular compartment (the mitochondria), and signal reduced in respiratory deficient cells and tissues. Manufactures' website shows western blot and immunocytochemistry analysis of the antibody and indicates it is a mouse monoclonal antibody reactive against the human protein. It has been cited in 191 publications, e.g. PubMed 33398350.
Anti-NDUFB8– correct sized protein in correct cellular compartment (the mitochondria), and signal reduced in respiratory deficient cells and tissues. Manufactures' website shows a western blots analysis of the antibody in lysates isolated from human, cow, rat and mouse heart. The antibody is cited in 111 studies (e.g. Alston et al. Pathogenic Bi-allelic Mutations in NDUFAF8 Cause Leigh Syndrome with an Isolated Complex I Deficiency, AJHG, 106 (1) 92-101 (2020); Sommerville EW et al. Instability of the mitochondrial alanyl-tRNA synthetase underlies fatal infantile-onset cardiomyopathy. Hum Mol Genet 28:258-268 (2019)).
SDHA - Manufactures' website shows a western blots analysis of the antibody in lysates isolated from wild type HEK93, SDHA KO HEK293, MCF7 and HepG2, in addition to human, bovine, rat and mouse heart mitochondria. The antibody was cited in 235 studies (Alston et al. A recessive homozygous p.Asp92Gly SDHD mutation causes prenatal cardiomyopathy and a severe mitochondrial complex II deficiency, Human Genetics 134, 869-879 (2015)).
Anti-GAPDH is a mouse monoclonal IgG1 κ GAPDH antibody; manufactures' website shows a western blots analysis of the antibody in lysates isolated from a variety of human cell types HMCF7, HeLa, C32, Jurkat, Hep G2 and BJAB. Cited in 2,286 publications.
Anti-VCL – A now discontinued antibody. Manufactures' website provides a datasheet that includes western blot and immunocytochemistry analysis of the antibody in human cells and lysates, and indicates it is a mouse monoclonal antibody reactive against the human, rat and hamster versions of the protein.
Anti-phosphorylated-AMPK-alpha, this rabbit monoclonal antibody detects a protein of the correct size, and as predicted for an ATP sensor, the phosphorylated version is only detectable when ATP levels are low in the cells. Manufactures' website shows Western blots analysis of the antibody in mouse C2C12 cells and immunohistochemical analysis of paraffin-embedded human breast carcinoma. The use of the antibody is cited in 1964 studies.
Anti-AMPK-alpha – the antibody raised in rabbit detects a protein of the correct size and the same molecular mass as the phosphorylated protein. Manufactures' website shows Western blots analysis of the antibody in lysates isolated from human and rodent cell lines. The use of the antibody is cited in 1396 studies.
Anti-PCNA- in our experiments this antibody raised in mouse detects a protein of the expected size in human fibroblast cell lysates, whose expression is markedly reduced in contact inhibited cells, where nuclear DNA replication is in abeyance. Manufactures'

website shows fluorescent western blot analysis of PCNA expression in HCT-116, Raji, HeLa, MOLT-4, NIH/3T3 and KNRK whole cell lysates. The use of the antibody is cited in 2622 studies.

Eukaryotic cell lines

Policy information about cell lines

Cell line source(s)	A549 adenocarcinoma cells were ATCC sourced; Myo-RD were kindly provided by Dr Vergani. Primary fibroblasts from controls and patients derived from UCL Biobank and Newcastle University Wellcome Center.
Authentication	A549 adenocarcinoma cells were not authenticated in this study. The Myo.RD cells were validated previously by differentiating them to myotubes, using F14 medium, 2% serum, 100nM 12-0-tetradecanoylphorbol-13-acetate (TPA) and 10 µg/mL insulin, and innervation to produce muscle fibres. Muscle differentiation was confirmed by qPCR detection of the muscle-specific isoenzyme creatine kinase (M-CK), and by the presence of fetal myosin, based on immunohistochemistry (PMID: 17626036). The fibroblasts of the patients were authenticated by the detection of mutant mitochondrial DNA. Control fibroblast lines had the expected properties and growth features and could be induced to enter quiescence (whereas immortal cell lines cannot).
Mycoplasma contamination	All cell lines were regularly tested for mycoplasma with a commercially kit and on no occasion tested positive. Additionally, mycoplasma is readily detected in DNA labelling experiments and was not seen.
Commonly misidentified lines (See ICLAC register)	None used

Human research participants

Policy information about studies involving human research participants

Population characteristics	The fibroblasts derived from 2 patients from 2 families. One patient experienced multiple stroke-like episodes with associated encephalopathy, which lead to a decline in cognitive and motor function, and progressive cardiomyopathy that contributed to premature death. The second patient presented with a pigmentary retinopathy, mild ophthalmoparesis in upgaze, and proximal muscle weakness.
Recruitment	Participants were recruited and diagnosed with suspected mitochondrial disease through accredited clinical diagnostic pathways.
Ethics oversight	The study design complied with all relevant regulations regarding the use of human study participants and was conducted in accordance to the criteria set by the Declaration of Helsinki. Written patient consent was obtained and the study was approved by the Queen Square Research Ethics Committee, London, (09/H0716/76) and NRES Committee North East-Newcastle & North Tyneside 1(16/NE/0267), UK.

Note that full information on the approval of the study protocol must also be provided in the manuscript.

Flow Cytometry

Plots

Confirm that:

- [x] The axis labels state the marker and fluorochrome used (e.g. CD4-FITC).
- [x] The axis scales are clearly visible. Include numbers along axes only for bottom left plot of group (a 'group' is an analysis of identical markers).
- [x] All plots are contour plots with outliers or pseudocolor plots.
- [x] A numerical value for number of cells or percentage (with statistics) is provided.

Methodology

Sample preparation	Primary human fibroblast cells were washed with PBS, trypsinized, centrifuged, resuspended in 300 µl of PBS and acquired, after treatments and labeling with probes.
Instrument	FACS Analyzer LSRFortessa 5 laser SORP, Becton-Dickinson
Software	Diva Software version 8, Becton-Dickinson
Cell population abundance	>100 000 live cells/experiment, no sorting applied, hence values apply to 100% of the live cell population analyzed in any given experiment.

Gating strategy

Fraction corresponding to cell debris (low FSC-SSC) was excluded from total cell count. Untreated TMRM-labeled single cells were used to define the gate for the TMRM positive cell population, while unlabeled and FCCP-treated (depolarized cells) were used to designate the gate for TMRM-negative population. These two cell populations made 100% of considered events, eg. cells falling outside TMRM positive or negative gates were not considered in calculating the percentages of each.

☒ Tick this box to confirm that a figure exemplifying the gating strategy is provided in the Supplementary Information.
