# Supplementary material for: 2-Deoxy-D-glucose couples mitochondrial DNA replication with mitochondrial fitness and promotes the selection of wild-type over mutant mitochondrial DNA
Source: Nat Commun. 2021 Dec 6;12:6997. doi: 10.1038/s41467-021-26829-0 (PMC8648849; doi:10.1038/s41467-021-26829-0)

Fig. 1g

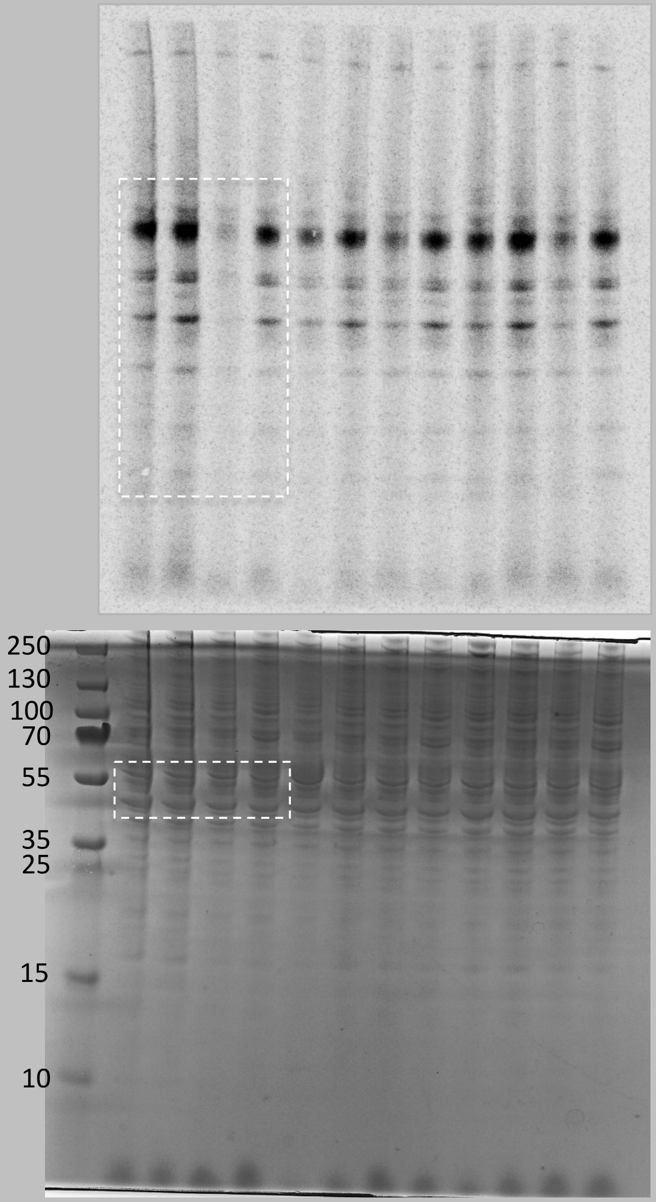

Fig. 1h

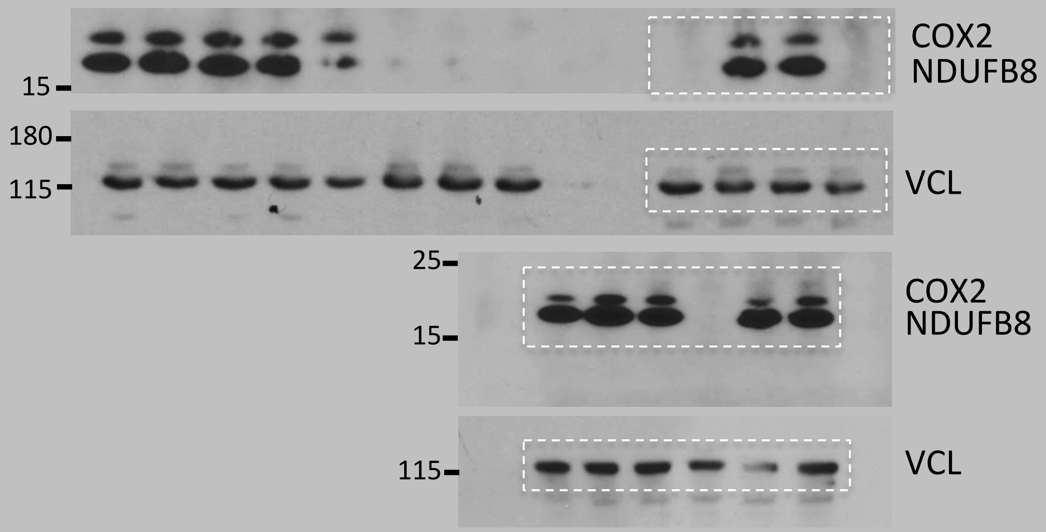

Fig. 1j

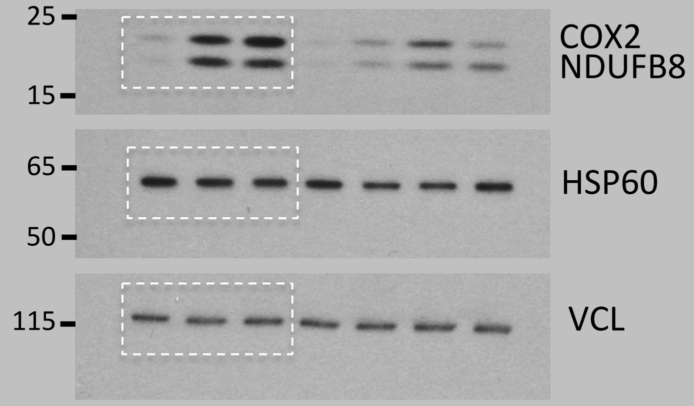

Fig. 3a

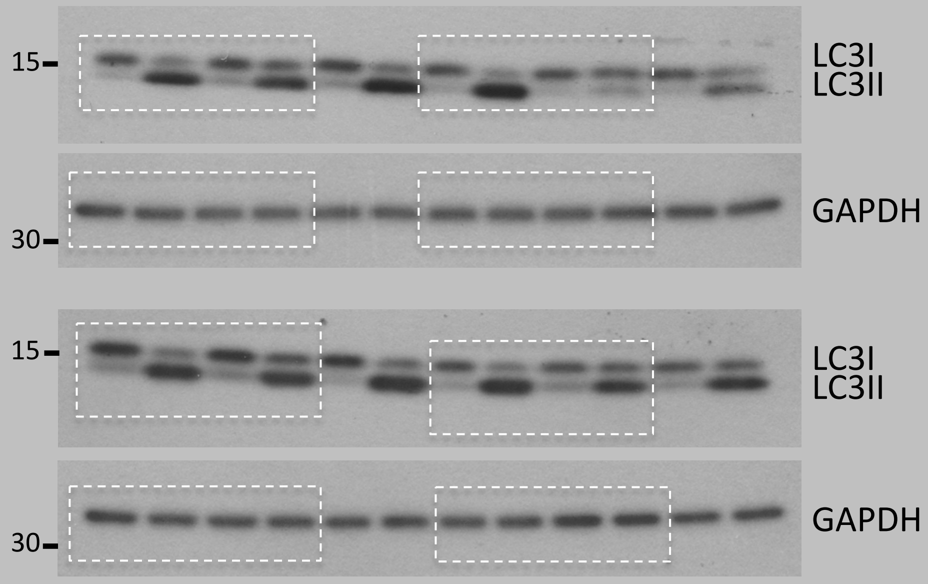

Fig. 3c

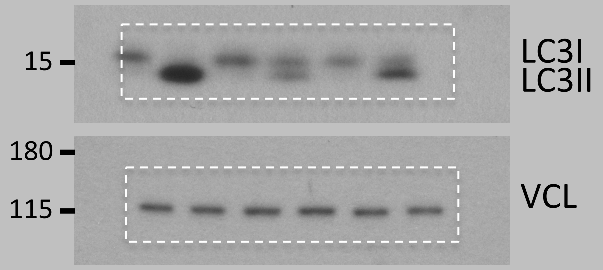

Fig. 3e

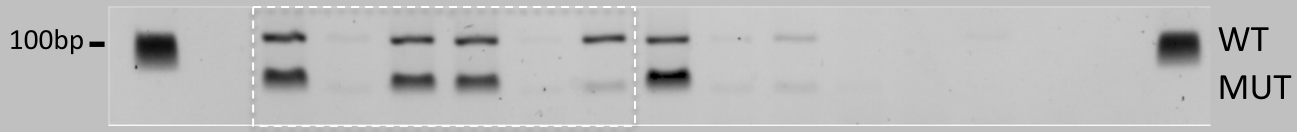

Fig. 4c

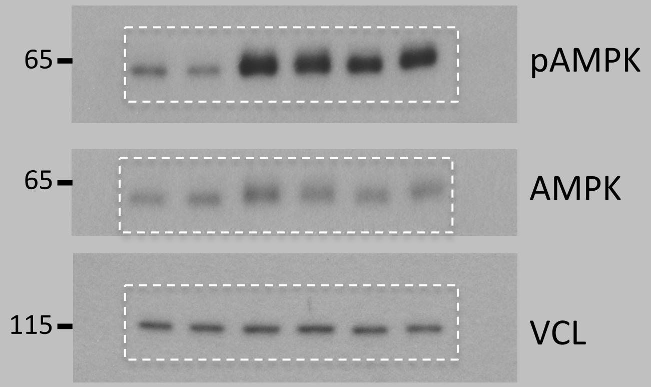

Fig. 4e

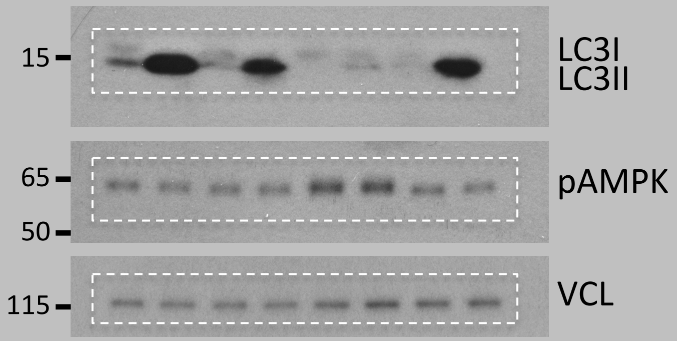

Fig. 6b

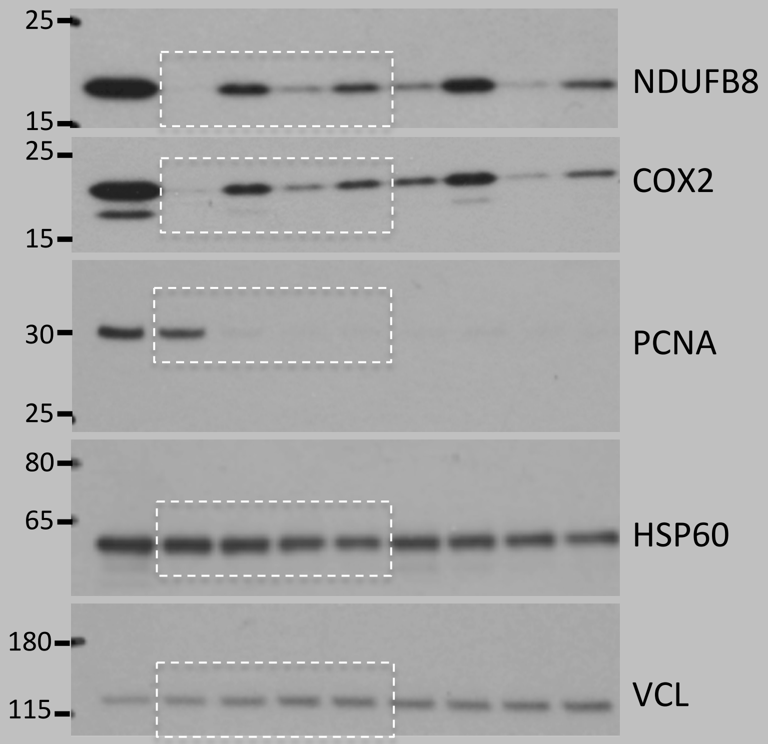

Supplementary Fig. 1d

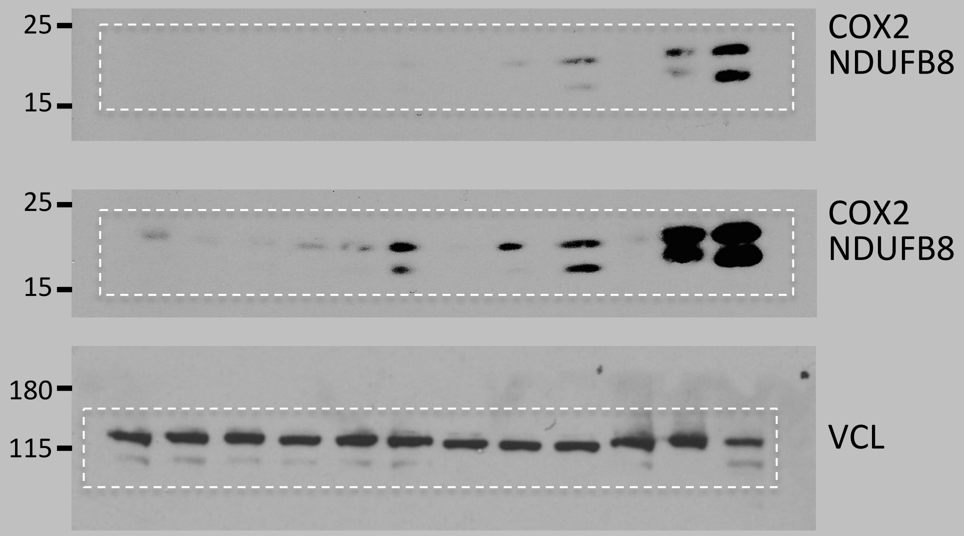

Supplementary Fig. 2d

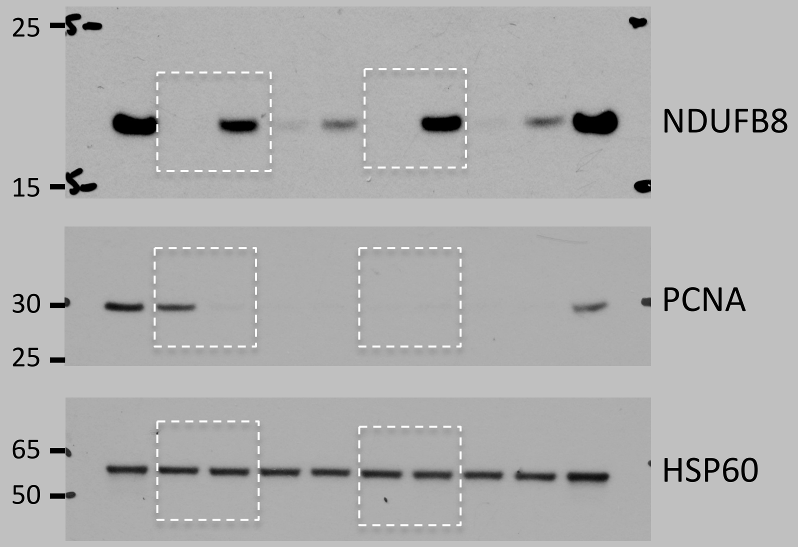

Supplementary Fig. 3a

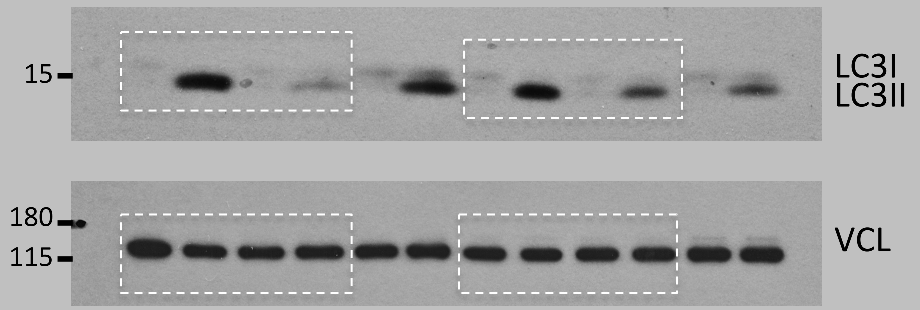

Supplementary Fig. 7b

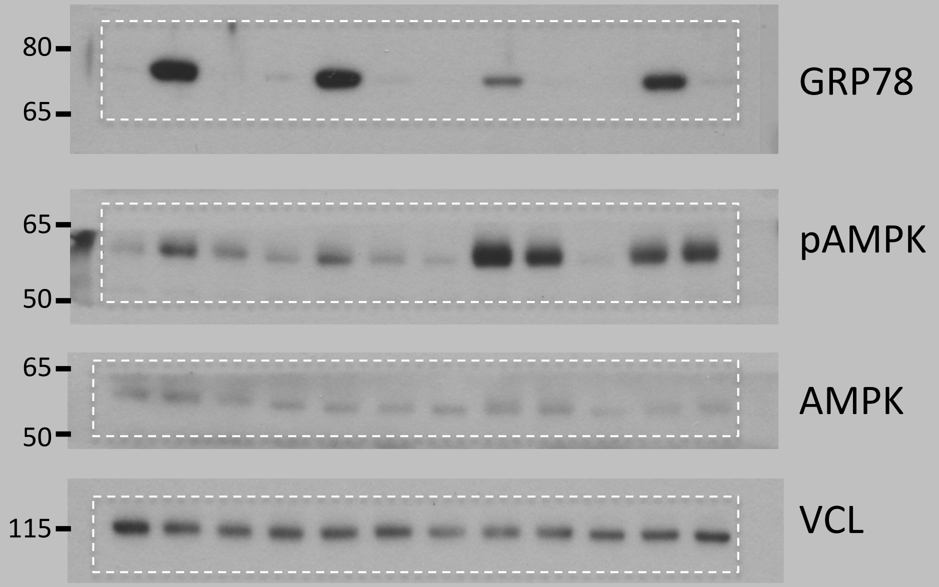

Supplement: Supplementary file 4 — Source Data. [file 41467_2021_26829_MOESM4_ESM.zip › Source of data_Western blots_NCOMMS-20-40547B.pdf]
